# Lead Drug Discover Strategies from Natural Medicines Based on Network Pharmacology

**DOI:** 10.18103/mra.v11i2.3559

**Published:** 2023-01-28

**Authors:** Shitang Ma, Jiafu Hou, Shijuan Liu, Fucheng Zhu, Peipei Wei, Chengtao Feng, Naidong Chen

**Affiliations:** 1 College of biological and pharmaceutical engineering, West Anhui University, Lu’an, China.; 2 Mudanjiang Medical University, Mudanjiang, China.; 3 Anhui University of Chinese Medicine, Hefei, China.

**Keywords:** lead drug discover, natural medicines, network pharmacology, screen, poly-pharmacology, multi-target

## Abstract

The need for therapeutics to overcome development of existing diseases research to discover new lead agents. In the face of public health challenges worldwide, natural medicines play a pivotal role in innovative lead drug discovery. Network pharmacology can easily construct complicated poly-pharmacology network based on lead compound, biological function, and bioactive target proteins, which meets the overall feature of natural medicines, and enable to elucidate the action mechanism at molecule-protein level with systematic view. In this work, we first summarized the recent progress delineating lead drug development and its interaction with natural medicines. Second, we focused on the relationship between natural medicines and network pharmacology. Additionally, we discussed current issues and potential prospects for the lead drug discover from natural medicines by network pharmacology. Further investigations should be focus on relevant structural analysis for biological experiment, also the dynamic and quantitative network development. In summary, it is a rational approach for innovative lead drug discovery, and with the development of structure and biology research, this approach makes it a very powerful method for the lead molecules in a high-throughput manner from a comprehensive and powerful special multi-compound to target protein/disease poly pharmacology network.

## Introduction

The identification and development of novel lead drugs play crucial roles as certain existing therapeutics can be less effective and/or intolerable side effects^1^. Lead drug discovery from Natural medicines (NMs) are essential in medicinal chemistry due to its structural diversity and relevant various biological activities^2^. NMs originate from natural sources including plants, marine organisms, and microorganism, etc. It continues to inspire the world in related interdisciplinary such as pharmaceutical chemistry with its unique advantages of additive or synergistic effects by simultaneously acting on multiple targets of disease pathway network^3^. Despite remarkable advances, the scourge of communicable and non-communicable diseases and the challenges of lead drug development with little or no side effects is still an enormous challenge at any time^4^. These terrible diseases such as COVID-19 continue to plague diverse populations worldwide with significant associated morbidities and mortalities^5^. There is urgent need for innovative lead drug discovery strategies that skew from the present strategies. A viable strategy will be to revert to NMs for answers since it has worked for drug discovery in the past decades^6^.

Due to the immense chemical and structural diversity with a wide variety of biological activity, most pharmacologically relevant lead drug and its derivative were designed, synthesized and evaluated its biological activity, including antitumor, anti-inflammatory, and antivirus. Notably, more than half of candidate drugs have been developed from NMs over the past decades. Antihepatitic drugs such as biphenyl diester (*Schisandra chinensis*)^7^, antimalarial drug artemisinin (*Artemisia annua*), and anticancer drug vinblastine (*Catharanthus roseus*) were all discovered and developed from NMs and are effective in treating these dreaded diseases. Advancements in relevant structural and bioinformatical analysis including molecular biology hold the key to lead a renaissance and achieves great progress in the field of new lead drug discovery. A variety of studies have shown that lack of adequate efficacy and clinical safety or toxicology are the two single most key reasons for lead drugs failures based on single target. On the contrary, NMs candidate leads increasingly being considered to derive safer and more effective therapeutics with desired clinical efficacy and lower toxicity. For example, vinblastine, an indole alkaloid from Catharanthus roseus, exerts anti-tumor effects by acting on a crosslinked target network composed by Tubulin alpha-1A chain, Tubulin beta chain, and Tubulin delta chain, etc.

Currently, many effective lead drugs such as artemisinin act via modulation of multiple targets rather than single protein^8^. This new appreciation of poly-pharmacology characteristic in the form of multi-component, multi-target and multi pathway has significant implications for tackling the two major obstacle of attrition in novel drug development^9^. With the advancements of biology, bioinformatics, and pharmacology, network pharmacology is considered to be a promising approach for lead discovery from NMs with holistic theory and a rich experience in multi-target therapeutics^10^. As a prominent technique with cost-effective characteristic, Network pharmacology offers a way of interpret about lead drug discovery from NMs with desired drug-like properties and/or unwanted off-target effects. It can made it possible to fully depict the overall perspective of the complex NMs with the potential to discover new lead drugs with novel therapeutic moieties for clinical use. Our recent investigations have demonstrated that network pharmacology procedure had extensive applications for lead drug screen. We once constructed the inflammation and related disease network^9^, and a series of compounds including emodin and ligustrazine were screened out with multi-target feature^11, 12^. In practice, many network-based computational and experimental studies were conducted, and several lead drugs with relevant target proteins were screened out. In detail, the general routing of network pharmacology to find lead from NMs are as follows: network construction, analysis and verification. Among which constructing a network firstly based on massive experimental or existing database searching data of compounds and related proteins is an easy beginning. There were various available databases and data resources related to small such as pubchem of NCBI, drugbank, and CHMIS-C, etc for small molecules, and genecard, uniport, etc for relevant target protein screen. Then screen out the key nodes of ligand compound and/or target and predict the key biological pathway via next network analysis^13^. Finally, network evaluation in form of in vivo/in vitro biological experiment^12^. Under this way lead drug would be screened out from NMs with characteristic of poly pharmacology. This usually applied to treat complex polygene diseases^14^.

Although it could easily screen out lead compound from a large amount data via a series of omics technologies based on the interaction between the ligand lead and its associated target protein, network pharmacology still has some limitations^15^. Firstly, the experimental or existing data of compounds from NMs is too incomplete compared with the disease gene network. Therefore, a more comprehensive database should be included and perfected information on the lead compounds from NMs. Secondly, no unified operating procedure. As different model algorithms were used in practice, it is necessary to select an appropriate model algorithm to ensure its accuracy for evaluation. Thirdly, its development was inseparable from the disease network. so massive experimental data of related disease in vivo or vitro is indispensable. Besides, the network pharmacology fails to characterize the interaction in quantitative manner. However, there is a dose efficacy relationship between the lead drug and disease. It is difficult to achieve the goal of quantitative rather than qualitative analysis based on network pharmacology methodology. Lastly, the occurrence and/or development of disease/drug interaction were also dynamic and individual different, the technology also fails to resolve this issue. Even the above unavoidable disadvantages exist in the network pharmacology, it indeed provides a novel poly pharmacology research strategy for screening lead compound from NMs.

In the future, with the development of relevant structural analysis for biological experiment and data analysis, it is expected to effectively solve the above limitations step by step. It would proceed the pace of lead discovery a huge step forward. Also, the dynamic and quantitative network development would be another tendency in the future. In a word, by effectively establishing a special multi-compound to target protein/disease poly pharmacology network, this approach makes it a very powerful method for the lead molecules in a high-throughput manner.

## Conclusion

In order to seek potential bioactive ligands for therapeutics of existing diseases, Network pharmacology provides a new horizon by mapping lead compounds into the disease-gene network. Relevant investigations showed the power of network pharmacology analysis in understanding lead discovery’s biological systems. By simultaneously embraces efforts to demonstrate the clinical efficacy and side effects/toxicity, network pharmacology provides a new horizon for lead drug discover from NMs with characteristic of multi-ligand compound, protein and pathway. Next, it is expected to create the foundation of the next paradigm in lead drug development with the advancements of structural biology for biological experiment and data analysis. Therefore, although the network pharmacology is still in its infancy, such a novel process would initiate new pathway and lead a probable revolution in the lead drug discovery field.
